# Supplementary material for: Autocatalytic chemical networks at the origin of metabolism
Source: Proc Biol Sci. 2020 Mar 11;287(1922):20192377. doi: 10.1098/rspb.2019.2377 (PMC7126077; doi:10.1098/rspb.2019.2377)
Supplement: Supplementary Methods, Figures and Tables [file rspb20192377supp1.pdf]

Supplementary Material

Autocatalytic chemical networks at the origin of metabolism

Proceedings of the Royal Society B; doi: 10.1098/rspb.2019.2377

Joana C. Xavier*, Wim Hordijk, Stuart Kauffman, Mike Steel, William F. Martin

*author for correspondence: xavier@hhu.de

Table of Contents

SUPPLEMENTARY METHODS 2

CATALYSIS-ANNOTATED METABOLIC NETWORKS 2
DETECTION OF MAXRAFS 3
RAF SETS 3
RAF ALGORITHMS 4
LUCA ENRICHMENT ANALYSIS 6
STATISTICAL ANALYSIS 6

SUPPLEMENTARY FIGURES 8

SUPPLEMENTARY TABLES 15

LEGENDS FOR SUPPLEMENTARY DATA 16

SUPPLEMENTARY REFERENCES 16

Supplementary Methods

Catalysis-annotated metabolic networks

All the reactions and the EC numbers they are linked to were retrieved from KEGG [1], along with their corresponding taxonomic annotations using the KEGG REST API (https://www.kegg.jp/kegg/rest/keggapi.html, accessed February 2018). The EC–reaction pairs were filtered by excluding reactions annotated only in eukaryotes. The corresponding chemical equations were then parsed to discard reactions involving molecular oxygen. Spontaneous reactions were parsed out of KEGG and added to the network with a fictional catalyst named "Spontaneous". Reactions catalyzed by enzymes that are not spontaneous and the enzymes of which do not use any cofactors were assigned the catalyst "Peptide". Reactions that equate synonymous cofactors were added with the generic catalyst "Pooling". Extensive curation was performed regarding catalysis rules, reaction reversibility, and amino acid production. The reversibility of reactions was parsed out of KGML files for KEGG pathways and manually–curated. The resulting set of reactions was then integrated with cofactor information from Uniprot [2] through the corresponding EC numbers. Of all EC numbers searched in Uniprot, 34% had at least one associated cofactor, 579 of which were EC numbers that involved more than one cofactor when parsed in a Boolean manner. Eighty-one unique cofactors were retrieved from Uniprot, which translated to 48 KEGG compounds or pools of catalytically equivalent cofactors linked to KEGG reactions through the EC numbers. Furthermore, cofactors directly participating in reactions (NADs, ATP, SAM, CoA, Cobalamins, Folates, Flavins and Quinones) were extracted from the reaction stoichiometry if not assigned as cofactors in Uniprot. All rules

were added to the network as additional parameters, and 66% of the final set of catalyzed reactions was assigned at least one small catalyst. The subsets for Met and Ace were obtained by crossing the genomic annotation of *Moorella thermoacetica* and *Methanococcus maripaludis* in KEGG with the previously built network, and with the addition of missing reactions that were present in corresponding manually–curated models [3,4]. The pipeline for the full procedure is shown in **Supplementary Material, Fig. S1**.

Detection of maxRAFs

All networks described above were tested for whether they contained maxRAFs with different food sets, which are described in the main text and available in **Supplementary Material, Table S1**. The fictional catalysts "Spontaneous" and "Pooling" were added in all tests, allowing for spontaneous reactions to always occur and synonymous cofactors to be equated. Pooling reactions that were part of the maxRAF were not accounted for in maxRAF sizes.

RAF sets

We define a *chemical reaction system* (CRS) as a tuple $Q = \{X, R, C, F\}$, where:

- $X = \{x_1, x_2, \dots, x_n\}$ is a set of molecule types.
- $R = \{r_1, r_2, \dots, r_m\}$ is a set of reactions. A reaction r is an ordered pair $r = (A, B)$ where $A, B \subset X$. The (multi)set $A = \{a_1, \dots, a_s\}$ indicates the reactants and the (multi)set $B = \{b_1, \dots, b_t\}$ indicates the products.
- $C \subseteq X \times R$ is a set of catalysis assignments. A catalysis assignment is a pair (x, r) with $x \in X$ and $r \in R$, denoting that molecule type x can catalyse reaction r.

- $F \subset X$ is a food set (i.e., molecule types that can be assumed to be available from the environment).

Given a CRS Q, a subset R' of R, and a subset X' of X, we define the *closure* of X' relative to R', denoted $cl_{R'}(X')$, to be the (unique) minimal subset W of X that contains X' and that satisfies the condition that, for each reaction $r = (A, B)$ in R',

$$A \subseteq X' \cup W \Rightarrow B \subseteq W.$$

Informally, $cl_{R'}(X')$ is X' together with all molecules that can be constructed from X' by the repeated application of reactions from R'.

Given a CRS $Q = \{X, R, C, F\}$ and a subset R' of R, R' is a *RAF set* if for each $r = (A, B) \in R'$

1. (Reflexive Autocatalysis): $\exists x \in cl_{R'}(F): (x, r) \in C$, and
2. (Food-generated): $A \subseteq cl_{R'}(F)$.

In other words, a subset of reactions R' is a RAF set if, for each of its reactions, at least one catalyst and all the reactants are in the closure of the food set relative to R' [5].

RAF algorithms

Given a CRS $Q = \{X, R, C, F\}$, an efficient (polynomial-time) algorithm exists for deciding whether Q contains a RAF set or not. It is presented formally in Algorithm 1.

Algorithm 1 RAF (X, R, C, F)

$R' = R$
change = true
while (*change*) **do**
    change = false
    Compute $cl_{R'}(F)$
    for all ($r = (A, B) \in R'$) **do**

if ($\nexists x \in \mathrm{cl}_{R'}(F) : (x, r) \in C \vee A \not\subseteq \mathrm{cl}_{R'}(F)$) **then**
$R' = R' \setminus \{r\}$
change = true
end if
end for
end while
Return R'

In plain words, starting with the full set of reactions R, the algorithm repeatedly calculates the closure of the food set relative to the current reaction set R' and then removes all reactions from R' that have none of their catalysts or not all of their reactants in this closure. This is repeated until no more reactions can be removed. If, upon termination of the algorithm, R' is non-empty, then R' is the unique *maximal* RAF set (maxRAF) contained in Q (i.e., a RAF that contains every other RAF in Q as a subset) [5]. If R' is empty, then Q does not contain a RAF set.

Computing the closure of the food set relative to the current reaction set R' is computationally the most expensive step in the RAF algorithm. It is presented formally in Algorithm 2. This closure computation algorithm, introduced in [5], is equivalent to the "network expansion" algorithm [6] used in [7].

Algorithm 2 ComputeClosure (F, R')

$W = F$
change = true
while (*change*) do
change = false
for all ($r = (A, B) \in R'$) **do**
if ($A \subseteq W \wedge B \not\subseteq W$) **then**
$W = W \cup B$
change = true
end if
end for

end while
Return W

A naive computational complexity analysis of the RAF algorithm gives a worst-case running time of $O(|X||R|^3)$. However, with some additional book-keeping (such as keeping track of all reactions that each molecule is involved in), this can be reduced. In fact, the average running time on a simple polymer-based model of CRSs turns out to be sub-quadratic [5].

LUCA enrichment analysis

The genetic families identified in [8] were mapped to KEGG orthologues, the corresponding EC numbers were retrieved and the reactions performed by these were listed and compared with the lists of reactions comprising the different networks, namely the global O_2-independent prokaryotic network; the maxRAF obtained with this network; maxRAFs obtained with the Ace and Met subsets; and the intersection of these.

Statistical Analysis

For pathway and cofactor enrichment analysis (**Fig. 3, 5A-B**), a contingency table was built for each comparison between a smaller network and the global network. The *p*-value refers to the probability of having at least as many reactions as seen (in pathway X or catalyzed by cofactor X) in a smaller network if we were to select a random pick of reactions the same size of that smaller network from the global network. For this, one-tailed Fisher tests (with Benjamini–Hochberg multiple test corrections) were used, and significance was considered for corrected *p*-values smaller than 0.05. A similar one-tailed Fisher test was

also used for calculation of enrichment in LUCA genes (**Fig. 5C**) and significance was considered for *p*-values smaller than 0.0001. All statistical analysis were performed in Python ver. 3.6.6 with the package scipy.stats. Network properties were calculated and visualizations were produced with Cytoscape [9] ver. 3.7.1.

Supplementary Figures

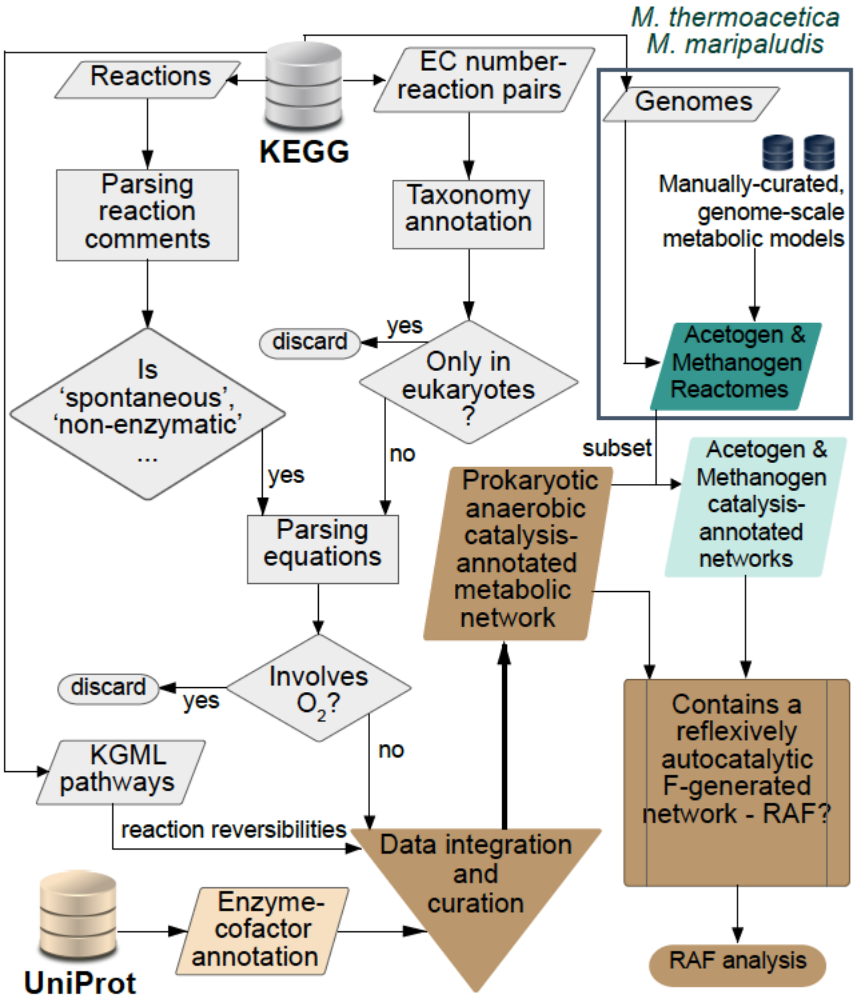

Fig. S1. Pipeline for reconstructing catalysis-annotated metabolic networks. Steps in grey include metabolic data only, steps in brown include catalysis rules, and steps in greens represent the inclusion of curated data from metabolic models of *Moorella thermoacetica* and *Methanococcus maripaludis*.

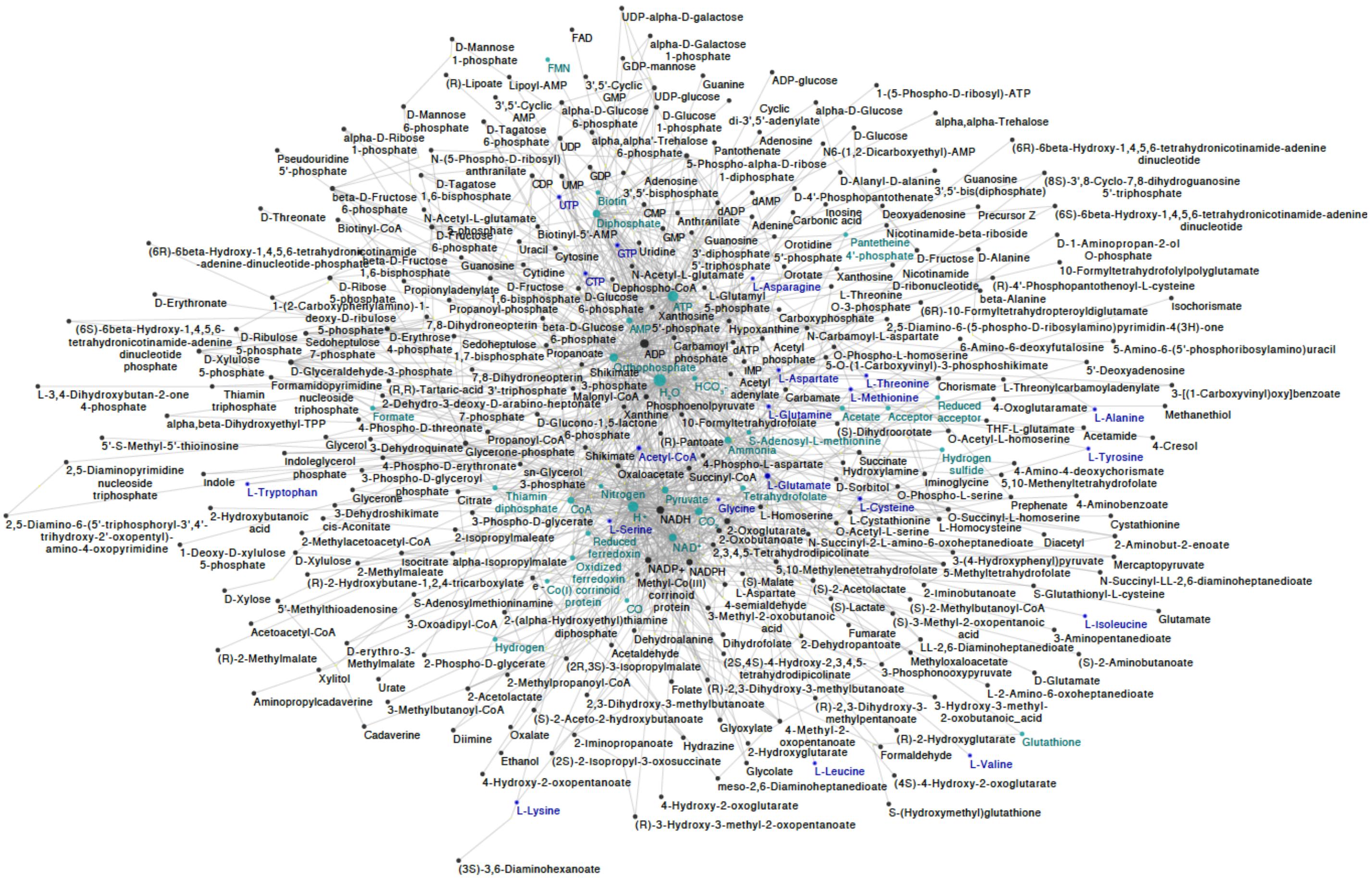

Fig. S2. MaxRAF obtained with the network of *Moorella thermoacetica*. Node size is scaled according to the degree, with food molecules highlighted in green and relevant products in dark blue (only metabolic interconversions are depicted; catalysis arcs are omitted for clarity). 'Acceptor' and 'Reduced Acceptor' are abstract redox molecules as represented in KEGG metabolism.

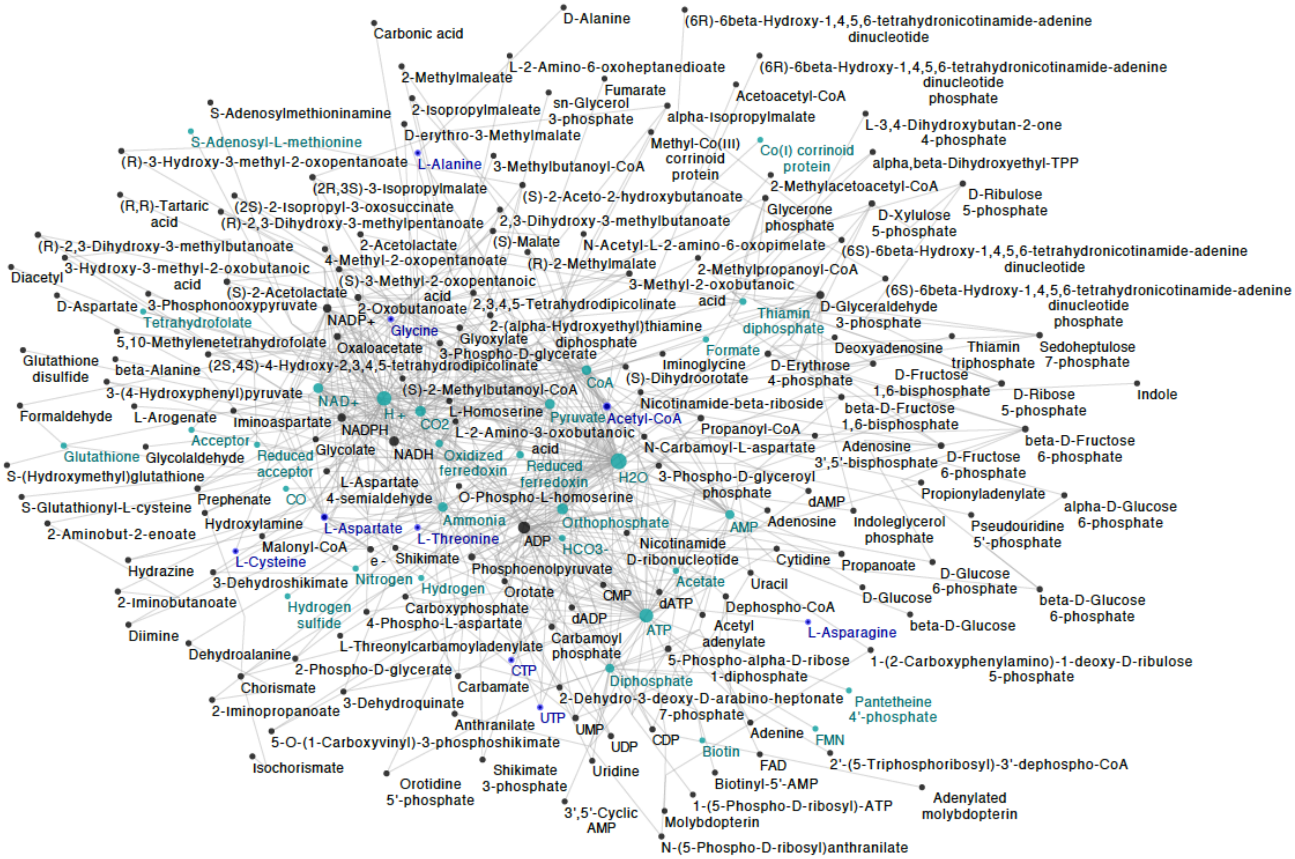

Fig. S3. MaxRAF obtained with the network of *Methanococcus maripaludis*. Node size is scaled according to the degree, with food molecules highlighted in green and relevant products in dark blue (only metabolic interconversions are depicted; catalysis arcs are omitted for clarity). 'Acceptor' and 'Reduced Acceptor' are abstract redox molecules as represented in KEGG metabolism.

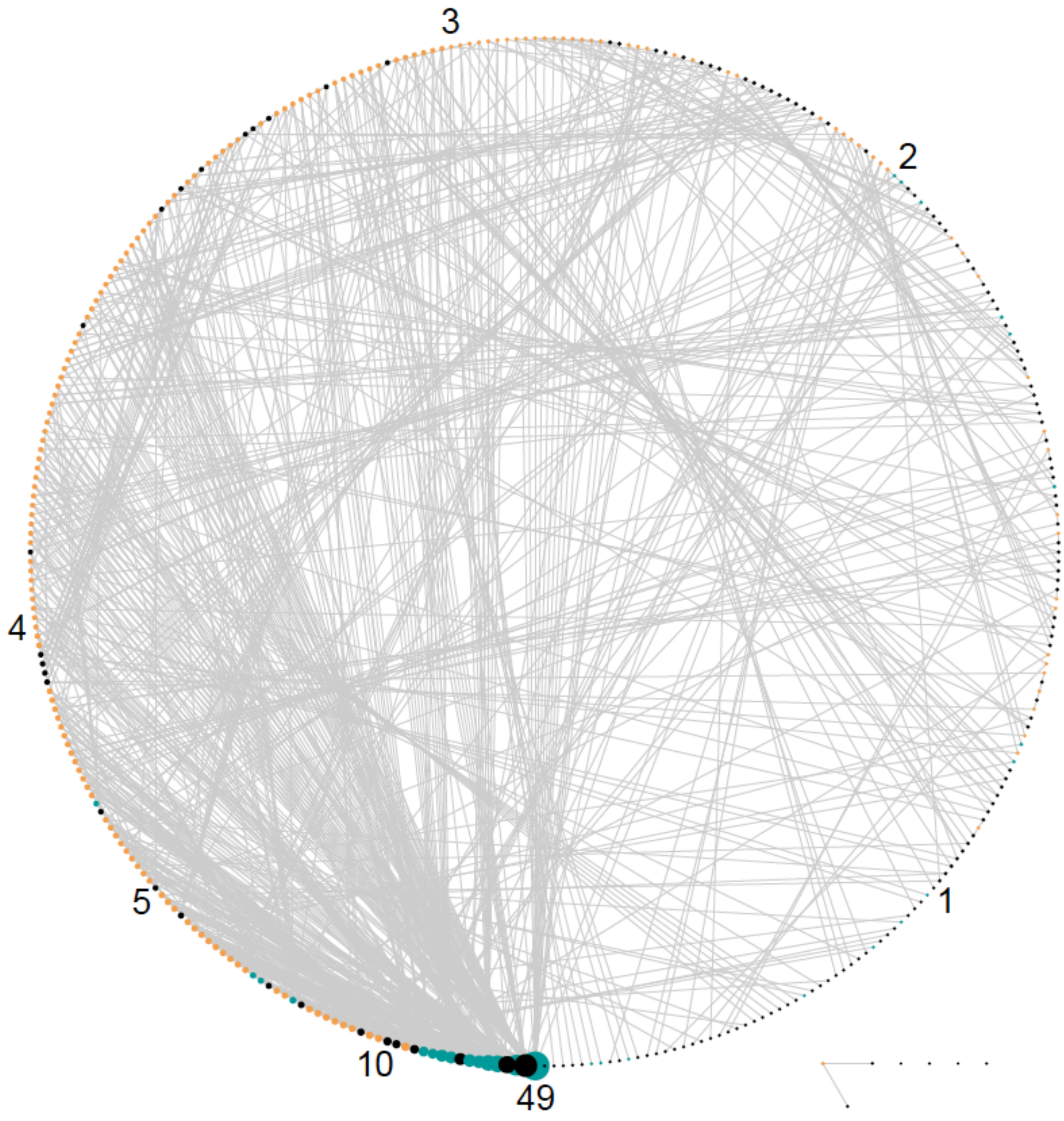

Fig. S4. Circular bipartite representation of the core autocatalytic metabolism at the origin of LUCA. Reactions (in orange) and metabolites (in green if food, grey for the rest) are represented as nodes. Nodes are sorted according to degree clockwise starting from the bottom; numbers show the degree at the respective position. ATP, the second most connected metabolite, can be removed from the food set without impact, therefore here is represented in black.

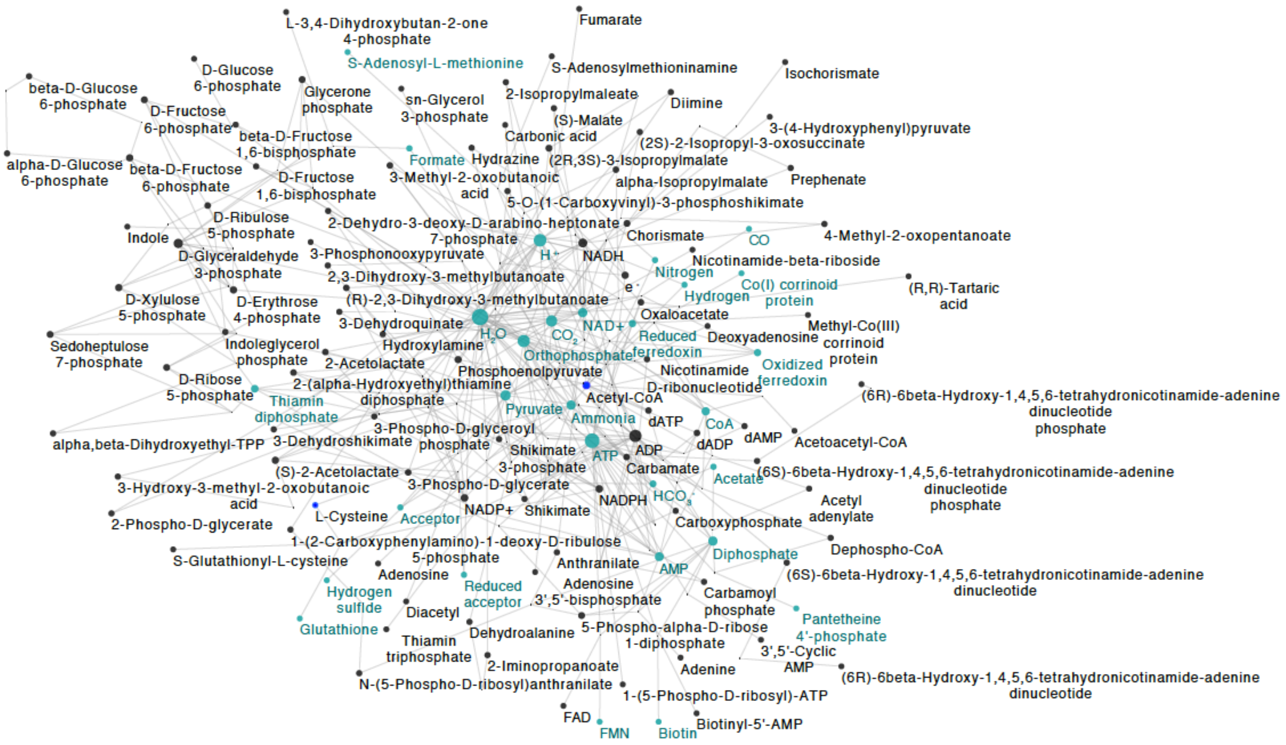

Fig. S5. MaxRAF obtained with the intersection of the networks of *Methanococcus maripaludis* and *Moorella thermoacetica*. Node size is scaled according to the degree, with food molecules highlighted in green and relevant products in dark blue (only metabolic interconversions are depicted; catalysis arcs are omitted for clarity). 'Acceptor' and 'Reduced Acceptor' are abstract redox molecules as represented in KEGG metabolism.

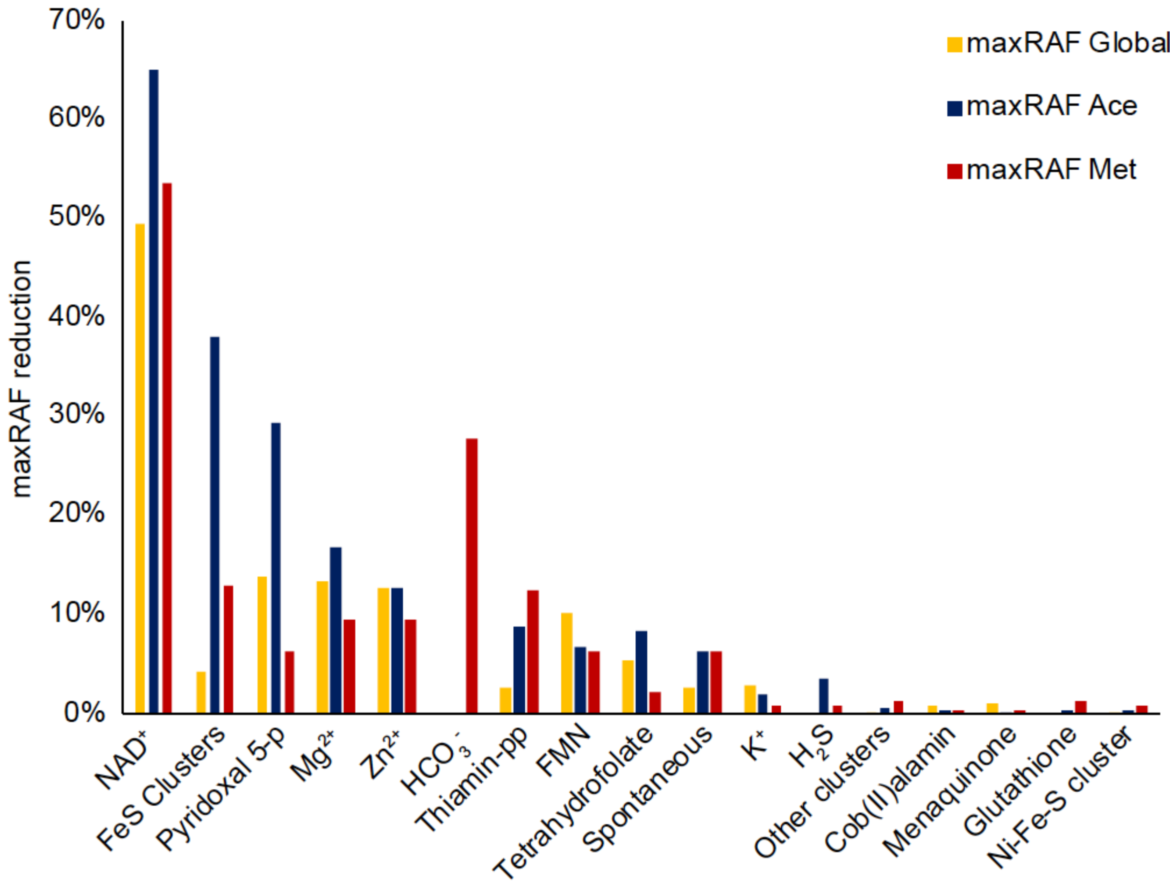

Fig. S6. Impact of removing single molecules from the food set with organic cofactors on the size of maxRAFs. The impact is shown as the reduction in size of the maxRAF (percentage of the initial network lost) when each molecule is removed from the food set with all organic cofactors, for the global prokaryotic O_2-independent network (yellow), *Moorella thermoacetica* (dark blue) and *Methanococcus maripaludis* (red). Cofactors with zero impact are not shown.

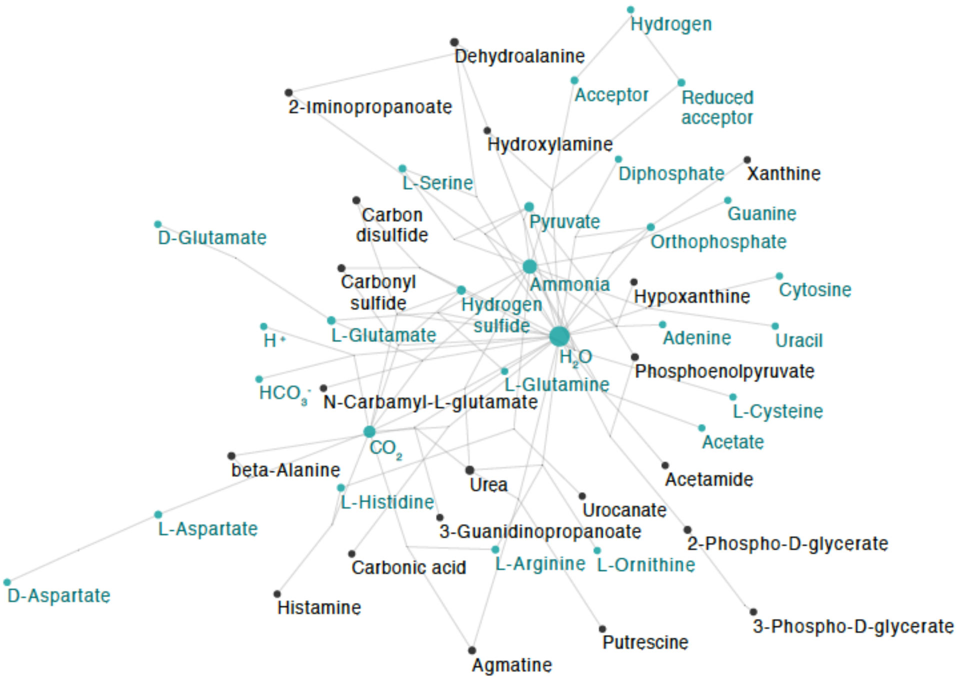

Fig. S7. MaxRAF obtained with amino acids and bases. The network represents the maxRAF obtained with the full prokaryote O_2-independent network with inorganic catalysts, abiotic compounds, all amino acids and bases but no organic cofactors added to the food set (only metabolic interconversions are depicted; catalysis arcs are omitted for clarity). Node size is scaled according to the degree, with food molecules highlighted in green. 'Acceptor' and 'Reduced Acceptor' are abstract redox molecules as represented in KEGG metabolism.

Supplementary Tables

Table S1. Composition of Food Sets used in predictions of maxRAFs in different metabolic networks and resulting maxRAF sizes.

ID	Description	Size	Content	maxRAF size		
				Global oxygen-free network	*M. thermoacetica*	*M. maripaludis*
**1**	Small-molecules + inorganic catalysts	39	H_2O, H_2, H^+, CO_2, CO, PO_4^{3-}, SO_4^{2-}, HCO_3^-, $P_2O_7^{4-}$, S, H_2S, NH_3, N_2, CO, all metals, Fe–S clusters, Ni–Fe–S cluster, other clusters, general acceptor, general reduced acceptor (donor), general metal, “Pooling”, “spontaneous”	8	4	4
**2**	**1** + abiotic organic carbon	43	**FS1** + Acetate, Pyruvate, Formate and Methanol	16	9	8
**3**	**2** + organic cofactors	68	**FS2** + FMN, Pyridoxal 5-phosphate, Thiamine diphosphate, NAD+, Molybdopterin, Cob(II)alamin, Pyrrolo-quinoline quinone, (R)-Lipoate, ATP, Biotin, Glutathione, Decylubiquinone, S-Adenosyl-L-methionine, Other quinones, Tetrahydrofolate, dipyrromethane, TTQ, 5-Hydroxybenzimidazolylcob(I)amide, AMP, Co(I) corrinoid protein, Pantetheine 4'-phosphate, Menaquinone, CoA, reduced ferredoxin, oxidized Ferredoxin	1335	394	209
**4**	**3** + Peptide	69	**FS3** + Peptide	2603	493	307
**5**	**2 +** aa and bases	68	**FS2** + all 20 amino acids, Adenine, Guanine, Cytosine, Thymine and Uracil	33	19	14

Legends for Supplementary Data

Dataset S1 (separate file). Metabolic networks annotated with catalysis rules. (A) Prokaryotic, O2-independent global metabolic network (B) subset network of Moorella thermoacetica (C) subset network of Methanococcus maripaludis.

Dataset S2 (separate file). Lists of reactions in all maxRAFs predicted for all networks in all food sets.

Dataset S3 (separate file). List and degree of metabolites in the primordial network shown in Fig.4 of the main text.

Dataset S4 (separate file). Input file with the global prokaryotic O2-independent network used to run the maxRAF algorithm. Food set with all small molecules, abiotic carbon and organic cofactors.

Supplementary References

1. Kanehisa M, Furumichi M, Tanabe M, Sato Y, Morishima K. 2017 KEGG: New perspectives on genomes, pathways, diseases and drugs. *Nucleic Acids Res* **45**, D353–D361. (doi:10.1093/nar/gkw1092)
2. UniProt Consortium T. 2018 UniProt: the universal protein knowledgebase. *Nucleic Acids Res* **46**, 2699. (doi:10.1093/nar/gky092)
3. Islam MA, Zengler K, Edwards EA, Mahadevan R, Stephanopoulos G. 2015 Investigating Moorella thermoacetica metabolism with a genome-scale constraint-based metabolic model. *Integr Biol* **7**, 869–882. (doi:10.1039/C5IB00095E)
4. Richards MA, Lie TJ, Zhang J, Ragsdale SW, Leigh JA, Price ND. 2016 Exploring hydrogenotrophic methanogenesis: A genome scale metabolic reconstruction of Methanococcus maripaludis. *J Bacteriol* **198**, 3379–3390. (doi:10.1128/JB.00571-16)
5. Hordijk W, Steel M. 2004 Detecting autocatalytic, self-sustaining sets in chemical reaction systems. *J Theor Biol* **227**, 451–461. (doi:10.1016/j.jtbi.2003.11.020)
6. Ebenhöh O, Handorf T, Heinrich R. 2004 Structural analysis of expanding metabolic networks. *Genome Inform* **15**, 35–45. (doi:10.11234/GI1990.15.35)
7. Goldford JE, Hartman H, Smith TF, Segrè D. 2017 Remnants of an ancient metabolism without phosphate. *Cell* **168**, 1126-1134.e9. (doi:10.1016/j.cell.2017.02.001)
8. Weiss MC, Sousa FL, Mrnjavac N, Neukirchen S, Roettger M, Nelson-Sathi S, Martin WF. 2016 The physiology and habitat of the last universal common ancestor. *Nat Microbiol* **1**, 16116. (doi:10.1038/nmicrobiol.2016.116)
9. Shannon P. 2003 Cytoscape: A software environment for integrated models of biomolecular interaction networks. *Genome Res* **13**, 2498–2504. (doi:10.1101/gr.1239303)
